# Extended Targeted and Non-Targeted Strategies for the Analysis of Marine Toxins in Mussels and Oysters by (LC-HRMS)

**DOI:** 10.3390/toxins10090375

**Published:** 2018-09-14

**Authors:** Inès Dom, Ronel Biré, Vincent Hort, Gwenaëlle Lavison-Bompard, Marina Nicolas, Thierry Guérin

**Affiliations:** 1 Laboratory for Food Safety, ANSES, Université Paris-Est, F-94701 Maisons-Alfort, France; ines.dom@anses.fr (I.D.); vincent.hort@anses.fr (V.H.); gwenaelle.lavison-bompard@anses.fr (G.L.-B.); marina.nicolas@anses.fr (M.N.); thierry.guerin@anses.fr (T.G.); 2 Agreenium, the French Agricultural, Veterinary and Forestry Institute, 75116 Paris, France

**Keywords:** marine toxins, LC-HRMS, targeted analysis, suspects screening, non-targeted analysis, method characterization

## Abstract

When considering the geographical expansion of marine toxins, the emergence of new toxins and the associated risk for human health, there is urgent need for versatile and efficient analytical methods that are able to detect a range, as wide as possible, of known or emerging toxins. Current detection methods for marine toxins rely on a priori defined target lists of toxins and are generally inappropriate for the detection and identification of emerging compounds. The authors describe the implementation of a recent approach for the non-targeted analysis of marine toxins in shellfish with a focus on a comprehensive workflow for the acquisition and treatment of the data generated after liquid chromatography coupled with high resolution mass spectrometry (LC-HRMS) analysis. First, the study was carried out in targeted mode to assess the performance of the method for known toxins with an extended range of polarities, including lipophilic toxins (okadaic acid, dinophysistoxins, azaspiracids, pectenotoxins, yessotoxins, cyclic imines, brevetoxins) and domoic acid. The targeted method, assessed for 14 toxins, shows good performance both in mussel and oyster extracts. The non-target potential of the method was then challenged via suspects and without a priori screening by blind analyzing mussel and oyster samples spiked with marine toxins. The data processing was optimized and successfully identified the toxins that were spiked in the blind samples.

## 1. Introduction

Marine toxins are natural compounds produced by certain microalgae that can contaminate a wide variety of marine species, including fish, crabs, or filter feeding bivalves (shellfish), such as mussels, oysters, scallops, and clams [1]. Different groups of toxins have been identified: saxitoxins (STXs), domoic acid (DA) and its isomers, tetrodotoxins (TTXs), okadaic acid (OA) and dinophysistoxins (DTXs), pectenotoxins (PTXs), yessotoxins (YTXs), azaspiracids (AZAs), ciguatoxins (CTXs), palytoxins (PLTXs) and ovatoxins (OVTXs), brevetoxins (PbTxs) and cyclic imines (spirolides (SPXs), gymnodimines (GYMs), pinnatoxins (PnTXs), pteriatoxins, prorocentrolides, portimine) [2]. These toxins are responsible for various biological activities and can exert deleterious effects on human health [3,4].

To protect human health from these toxigenic compounds and to avoid food poisoning, the presence of certain toxins in food destined for human consumption is regulated within the European Union (EU) [5,6] and is submitted to monitoring programs. These regulations clearly mention the toxins to monitor, the thresholds that should not be surpassed and the methods of analysis. Biological assays using mice and rats were prescribed as reference methods for certain toxins but are subject to controversy due to ethical issues and their lack of specificity [7]. To perform official monitoring of some toxins, such as saxitoxins and lipophilic toxins, chemical methods replace animal bioassays [8,9]. This is the case of liquid chromatography coupled with mass spectrometry (LC-MS). LC-MS methods are reported for the analysis of several toxin families in shellfish either individually or together [10,11,12,13,14,15]. Target monitoring approaches are fit for regulatory purposes as they achieve good sensitivity and specificity. These methods are based on a targeted screening that only seeks to find a short list of predetermined compounds, while missing all other toxins that could be present in the sample. To be fully integrative with respect to consumers’ safety, monitoring programs should be able to detect the appearance of so called “emerging toxins”. The latter include newly discovered toxins/toxin analogues, as well as the detection of known toxins in areas where they had not been previously described. Methods using high resolution mass spectrometry (HRMS) have been recently developed and used for the analysis of a larger panel of marine toxins in a single run [16,17,18]. The HRMS technology enables reliable analysis with excellent specificity and selectivity that are necessary to resolve the interference from complex matrices, such as mussels and oysters [19,20,21]. Besides, this technology offers new monitoring capabilities such as retrospective analysis and the possibility to move from targeted to non-targeted analysis allowing the identification of “unknowns”. Yet, the non-targeted analysis is a very challenging task, as it requires extensive processing of the generated dataset. To render these data meaningful, multistep strategies using chemometric tools are required before the final identification of a specific signal among a forest of interfering signals.

While there are several studies in the literature regarding the characterization and validation of targeted methods for the analysis of marine toxins in different matrices, both in low and high resolution [11,15,16,18,22,23,24,25], there are no studies presenting an appropriate characterized strategy for the non-targeted approach in the field of marine toxins. Only a few papers, inspired from the metabolomics approaches dealing with the analysis of environmental samples (wastewaters), addressed this challenge [26,27,28,29,30,31].

**Figure 1:**
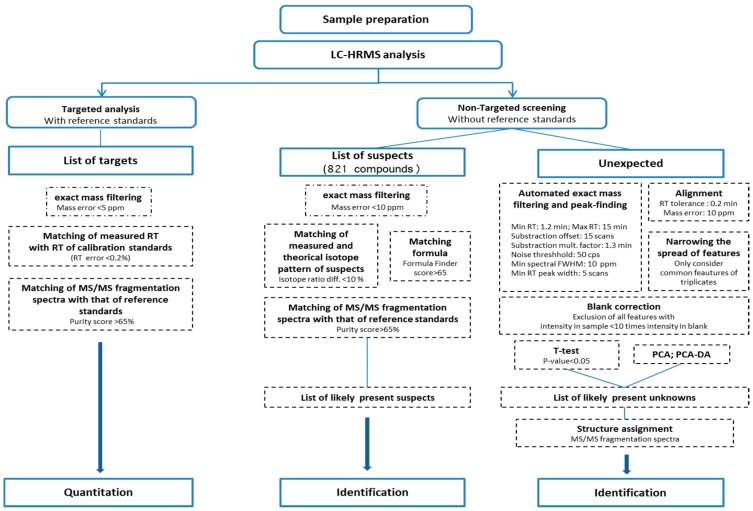
Processing workflow with optimized parameters for (**1**) quantitative targeted analysis, (**2**) suspect screening and (**3**) non-targeted screening of unknowns using liquid chromatography coupled with high resolution mass spectrometry (LC-HRMS) (adapted from Krauss et al. [26] and Nürenberg et al. [28]).

This paper describes the implementation and the characterization of an LC-HRMS method for the analysis of different toxins with an extended range of polarities, including lipophilic toxins and domoic acid, by the accurate measurement in MS and MS/MS modes in a single run while using a hybrid quadrupole time of flight mass spectrometer (QTOF). The expression “method characterization” should be understood throughout the manuscript as the assessment of some performances of the method but is different from a method validation, which is a more extensive and complete process. The method developed relies on a workflow (Figure 1) combining both targeted and non-targeted analysis composed of three approaches; (1) targeted screening similar to low resolution MS where reference standards are used to search for the compounds of interest; (2) suspect screening that consists of querying a database/library including an exhaustive list of suspect compounds for which reference standards might not be available; and, (3) non-targeted screening performed without a priori, thus, without reference standards or suspects to identify unexpected compounds [26,28]. First, suitable chromatographic conditions are chosen for the separation of the selected toxins with a broad range of polarities. The performance of the targeted quantitative analysis is assessed as a prerequisite for the non-targeted analysis. A multistep specific data filtering strategy from data acquisition to the final tentative identification of interesting ions is established and optimized by reducing the size of the search space. The general workflow for suspects and without a priori screening is tested and critically evaluated through the analysis in blinds of shellfish samples that are spiked with marine toxins.

## 2. Results

### 2.1. Targeted Analysis

#### 2.1.1. Method Development

The method was developed to analyze a large range of lipophilic and relatively polar toxins. A total of 18 toxins for which certified standard solutions were available were analyzed either in positive or negative ionization mode; azaspiracids 1, 2, and 3 (AZA1–3), pectenotoxin 2 (PTX2), okadaic acid (OA), dinophysistoxins 1 and 2 (DTX1 and 2), yessotoxin (YTX), homo-yessotoxin (hYTX), 13-desmethyl spirolide C (SPX1), pinnatoxin A and G (PnTX-A, PnTX-G), gymnodimine A (GYM), 13,19-didesmethyl Spirolide-C (13,19-didesMeC), 20-methyl spirolide-G (20-meG), domoic acid (DA), and brevetoxins 2 and 3 (PbTx-2 and PbTx-3).

Regarding all the toxins analyzed in ESI^+^ (GYM, SPX1, 13,19-didesMeC, 20-meG, PnTX-A and G, AZA1 to -3, PTX2, PbTx-2, and -3), protonated molecules [M + H]^+^ were detected except for PTX2, for which the [M + NH_4_]^+^ adduct was chosen as the characteristic ion. Concerning ESI^−^, the deprotonated form [M − H]^−^ was detected for OA, DTX1 and 2, YTX and hYTX. DA was detected in both ionization modes, but a better sensitivity was observed in ESI^−^. The deprotonated form [M − H]^−^ of DA (*m*/*z* 310.12961) was used for identification and quantitation purposes.

Since working in high resolution, toxin identification was mainly based on the exact mass of each molecule. Thus, the mass accuracy (expressed in ppm) was determined while using the suspects list. The corresponding standard deviations (SDs) of the masses for the intra-day and inter-day precision ranged from 0.4 to 1.7 ppm (Supplementary Material Figure S1). These results show good stability in mass measurements in the current analytical conditions.

**Figure 2:**
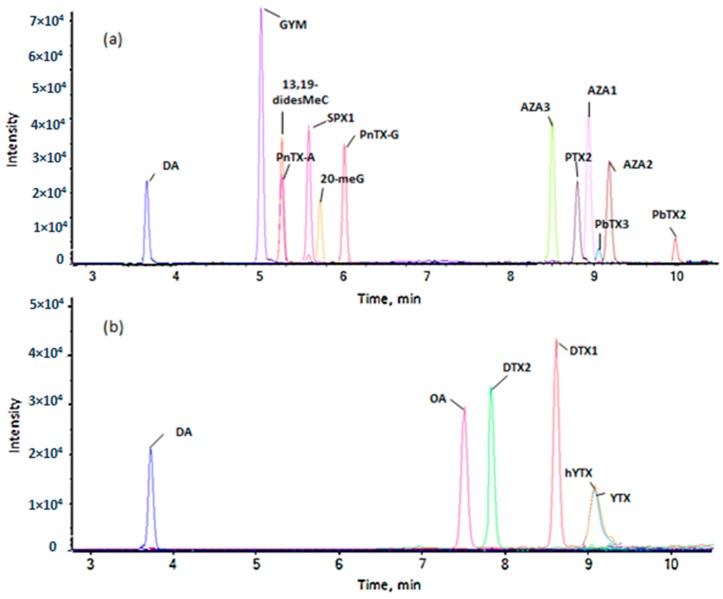
Separation of the different marine toxins in the optimized chromatographic conditions, analyzed on a 5600 quadrupole time of flight mass spectrometer (QTOF) (**a**) in positive ionization and (**b**) negative ionization mode.

Optimized LC conditions while using the C18 HSS T3 column allowed for a good separation of most of the toxins analyzed in both ionization modes (Figure 2). Chromatographic resolution was mostly important in the case of isobaric toxins (OA/DTX2), for which satisfactory separation was achieved. The inter-day and intra-day deviations in retention time (RT) did not exceed 0.2% over the course of the study, and were therefore negligible for all the targeted toxins.

A target compound list was created, including information, such as exact mass, adduct, and retention time, of all toxins analyzed to be used for quantitation (Supplementary Material Table S1). A library including the MS and MS/MS spectra of the available standards was also created for further confirmation purposes.

#### 2.1.2. Characterization Study of the Quantitative Method

Several criteria were investigated to evaluate the suitability of the quantitative method. The linearity of the calibration curves was verified by a correlation study. All of the determination coefficients (*R*^2^) were above 0.99 for both solvent and matrix-matched calibration curves (Supplementary Materials Table S2). Specificity was assessed by analyzing and comparing non-spiked and spiked blank mussel and oyster samples. Results showed that all the toxins were only detected in the spiked samples at specific retention times. The high resolution in MS analysis also contributed to the specificity of the method and confirmed that toxins in real shellfish samples can be screened and clearly identified.

The sensitivity of the method was evaluated by the assessment of LODs and LOQs in MeOH and two different matrices, typically mussels and oysters (Table 1). Good sensitivity was achieved for the 14 toxins included in the characterization study. Using mussel extract, limits of quantification (LOQs) were comprised between 2.0 µg/kg (GYM) and 8.9 µg/kg (OA). DA had a higher LOQ (30 µg/kg). LOQs determined in the oyster extract were slightly lower for all toxins compared to the sensitivity in mussel extract, except for PnTX-G, DTX1, and DA. The lowest LOQs were achieved in MeOH, in the absence of matrix, with values ranging from 1.1 to 26 µg/kg for GYM and DA respectively.

Regarding YTXs, LOQs were relatively higher than expected when compared to a triple quadrupole detector, but it can still be considered as acceptable. Brevetoxins were the least sensitive among the studied toxins in the presented conditions. A change in the mobile phase B from ACN to MeOH (keeping the rest of the composition the same as reported in the materials and methods section) allowed for a much better sensitivity for PbTx-2 and 3 (by a factor of 6–10). As most of the toxins analyzed responded well in the ACN mobile phase, the authors decided to keep this composition and evaluate the method for the 14 toxins with the lowest LOQs, excluding YTXs and PbTxs.

**Figure 3:**
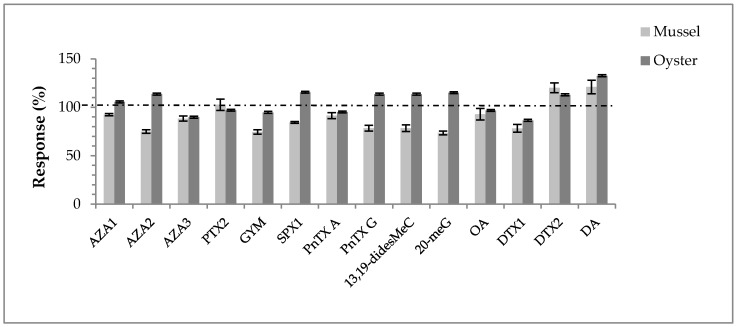
Matrix effects observed for the tested toxins in mussel and oyster extracts. The dashed line representing the 100% response corresponds to the results obtained in MeOH, used as a reference.

Matrix effects for mussel and oyster extracts were determined by a comparison of the mean slope of MeOH calibration curves (*n* = 3) to those of the matrix-matched calibration curves (*n* = 3). Observed effects can be described either as signal enhancement (responses >100%) or signal suppression (response < 100%). Concerning the mussel extract, 11 of the toxins tested exhibited an ion suppression effect ranging from −8% for AZA1 to −27% for 20-meG (Figure 3). No matrix effect was observed for PTX2, while DTX2 and DA were responsible for ion enhancement with a +20% signal gain in mussel matrix. Concerning the oyster matrix, ion enhancement was observed for eight toxins (8 out of 14) with values generally <+20%, ranging from +6% (AZA1) to +15% (for SPX1 and 20-meG), except for DA (+33%). The six remaining toxins showed ion suppression ranging from −3% (PTX2) to −13% (DTX1). Matrix effects are globally considered as satisfactory and no correction factors were applied for quantitation.

The accuracy of the method was verified by measuring the recoveries from blank mussels spiked at two concentration levels, six replicates for each fortification level. Following the EU Commission decision 2002/657/EC as a guideline, the proposed method was found to be accurate with satisfactory recoveries ranging from 86% to 110% for the low spike level and from 95% to 106% for the high spike level (Table 2). These results confirm that the extraction method is well adapted for all of the toxins analyzed. This shows that discriminating purification steps, which might be a limitation for non-targeted analysis, can be avoided. The precision of the assay, reflected by the repeatability and within-laboratory reproducibility, was investigated by means of the relative standard deviation (%RSD). The %RSD values that were obtained for intra-day (RSD_r_) and inter-day variations (RSD_R_) ranged from 1.3% to 13.7% and from 1.7% to 19.8%, respectively, depending on the toxins (Table 2). The precision was generally better for the high spike levels (120, 240 or 720 µg/kg). Results are within the acceptance criteria (<20%) demonstrating that the proposed method is considered as precise and it could be adopted for quantitative analysis.

#### 2.1.3. Application

**Figure 4:**
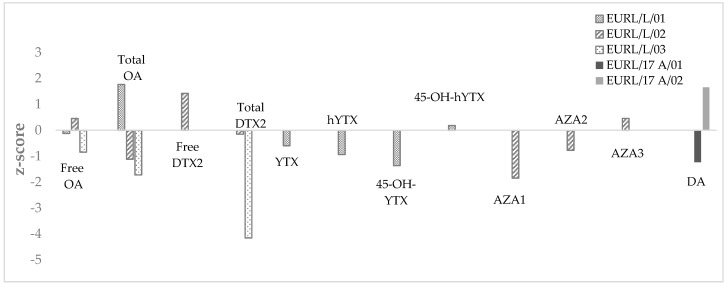
Z-scores obtained after analyzing, by LC-HRMS, the five samples provided by the EURLMB as part of a proficiency testing scheme for lipophilic toxins and DA.

Homogenates of naturally contaminated mussel tissues (*Mytilus* spp.) containing lipophilic toxins or DA proposed by the European reference laboratory for marine biotoxins (EURLMB) as part of proficiency testing schemes were analyzed by LC-HRMS. The results highlighted a very good agreement with the assigned values (Figure 4). All the lipophilic toxins (OA, YTX, hYTX, 45-OH-YTX, AZA1 to 3) were correctly identified and quantified (Z-score values comprised between −2 and +2), except for total DTX2 in sample EURL/L/03 (Z-score value of −4.2), due to its concentration around the LOQ. Samples containing DA were quantified correctly and the results are within acceptable Z-score limits (|z| < 2). These results confirm that the method developed is suitable for the quantification of both lipophilic toxins and DA in naturally contaminated shellfish samples.

The method developed has demonstrated good performances for the identification and quantification of many toxins belonging to different groups with a wide polarity range, proving that it is fit for targeted analysis. The method was fully characterized for the studied toxins, except for YTXs and PbTxs.

### 2.2. Non-Targeted Analysis

#### 2.2.1. Evaluation of the Suspect Screening Approach

Criteria for the identification and confirmation of suspects needed to be carefully chosen to minimize the risks for both false-positive (features erroneously identified as peaks of interest) and false-negative results. Different parameters were selected and optimized for accurate mass matches through the suspects list. A minimal intensity of 1000 counts was required for a minimal area representative for an actual peak. An imposed minimum signal-to-noise ratio was set to 6:1 (comprised between the accepted criterion defining LOD (S/N 3) and LOQ (S/N 10)) as peak picking criterion to define the decision limit.

Identification criteria for suspect screening were determined from triplicate injections of reference standards. For each targeted compound, the software displays different parameters: mass error, formula finder score, isotope match, etc. The worst scores that were obtained in these experimental conditions were chosen and reported as confidence settings for the identification of compounds via the suspect list. Thus, it was empirically determined that selecting compounds with a formula finder score above 65 would reduce the number of potential false positives and reach the minimum number of false negatives. A 10 ppm mass error and 10% isotope ratio difference were selected as the best compromise for the suspect screening as it weeds out non-specific formula matches without losing too many matches for compounds that have peak distortions due to their large peak areas or detector saturation. Choosing appropriate values for different filtering criteria is the key step for suspect screening strategies.

The suspect screening strategy was tested on spiked mussel and oyster samples that were analyzed in ESI^+^. Comparable data were obtained for the different matrices, therefore only results relative to oyster samples are presented. After applying our final traffic light color-coding filtering step (retaining only ‘green light’ features) based on the chosen criteria mentioned above, 15 suspect compounds out of a list of 821 were first identified as toxins potentially present in the analyzed samples. For verification purposes, the presence of actual chromatographic peaks and coherence in retention times were checked for each compound in the replicate injections (*n* = 3). This second step led to the elimination of five candidates that were either not present in all three injections (and therefore considered as false positive features) or not fulfilling the criteria of an actual peak. This step led to a list of 10 candidates, including PnTX-A, PTX2, GYM, SPX1, and two of its isobaric analogues, AZA1 and three of its isobaric analogues. Since many molecular formulas can give the same accurate mass, it is only through the MS/MS fragmentation data that reliable identification of the peaks was achieved. A final confirmation step was then applied by means of the comparison of MS^2^ spectra acquired with theoretical fragment spectra derived from mol files that were obtained from ChemSpider or PubChem databases (Supplementary Materials Figure S2). All the experimental spectra showed good correlation with theoretical fragments (>70%); SPX1 and AZA1 could be identified as the analogues present in the analyzed samples. An exception was observed for GYM, with only 20% matching fragments. The software automatically attributed the most intense peak present in the extracted chromatogram (XIC) as corresponding to the exact mass of the selected compound in the suspect list. The XIC of GYM was then checked visually and a second less intense peak was present at a different retention time (5.1 min) in the three different replicates. This peak was then manually selected and the correlation between the empirical and theoretical fragmentation spectra checked again, and this time the authors had a 100% match. This shows the importance of keeping a critical mind when handling results automatically generated and not taking them for granted until they have been verified.

#### 2.2.2. Evaluation of the Non-Targeted Screening Approach

Data that were generated from the blind test (Section 5.7) were processed while using two different options as part of the non-targeted screening; the first one consisted of comparing contaminated and non-contaminated samples pairwise while using a *t*-test to identify features only present in the contaminated samples. This option required that a non-contaminated sample with the same characteristics (elemental composition, species, location, et al.) as the contaminated one be available. The second option was to perform a multivariate analysis while using another statistical test, a principal component analysis (PCA).

##### Pairwise Comparison: *t*-Test Results

A *t*-test was carried out on the 5000 most intense features (*m*/*z*) and the data were then classified according to the increasing values of the *p*-value. Only ions with a *p*-value below 0.05 were investigated. Among all the data evaluated, only 100 to 150 ions (depending on the concentration level) out of the 5000 generated had a significant *p*-value below 0.05.

Regarding this test, the aim was to check whether the *t*-test is an appropriate tool allowing for the identification of the molecules of interest among the 100–150 ions selected based on their *p*-value. Selected ions were manually reprocessed while using PeakView^®^ to confirm that they were (1) corresponding to actual peaks (2) absent from the blank control samples and (3) present in the three replicates. Features not responding to these criteria were eliminated; this step allowed for reducing by half the list of ions of interest. Concerning both mussel and oyster matrices, the authors could identify clearly the supplemented toxins among the final list of features considered as responsible for the significant differences between contaminated and blank samples.

Table 3 shows the *p*-values obtained corresponding to each toxin for the six studied levels of contamination; SPX1 was the only toxin with a *p*-value below 0.05 for all six concentration levels in the mussel matrix, meaning that significant differences could be observed between the contaminated and non-contaminated samples for all six levels. Inversely, PTX2 had *p*-values below 0.05 only for the three most concentrated levels, typically L4–L6. Significant differences were observed between the blank mussel sample and the contaminated ones for AZA1 and PnTX-A from level 3 onward. Regarding the case of GYM, five out the six concentration levels had *p*-values below 0.05; the first concentration level was the only one that did not show a significant difference between the contaminated and non-contaminated samples. This illustrates the difference between the analytical determination limits inherent to the targeted method performances presented before and the discriminating power of the statistical tool. That proves that it is equally important to develop a sensitive method and to set up the adequate workflow able to pick up the signals of interest in a forest of features.

##### Multivariate Analysis: PCA and PCA-DA Results

Initially, an unsupervised PCA test was carried out on the data that were generated after the analysis of the MeOH and matrix-matched samples. The representation obtained following this first PCA test and the PC1/PC2 scores plot showed the presence of three different clusters corresponding respectively to solvent, oyster and mussel extracts (Supplementary Materials Figure S3). This shows that components **1** and **2** reflect the variability related to matrix ions. This distribution is not surprising as matrix ions are predominant when compared to the ions representative of the compounds of interest. The study of the other components did not reveal any clusterization based on the presence or absence of toxins either.

To overcome or reduce the impact of the matrix variability that was preponderant during the first test, the authors carried out a supervised PCA–DA, which allowed for a definition of the samples of mussels and oysters as belonging to the same group.

**Figure 5:**
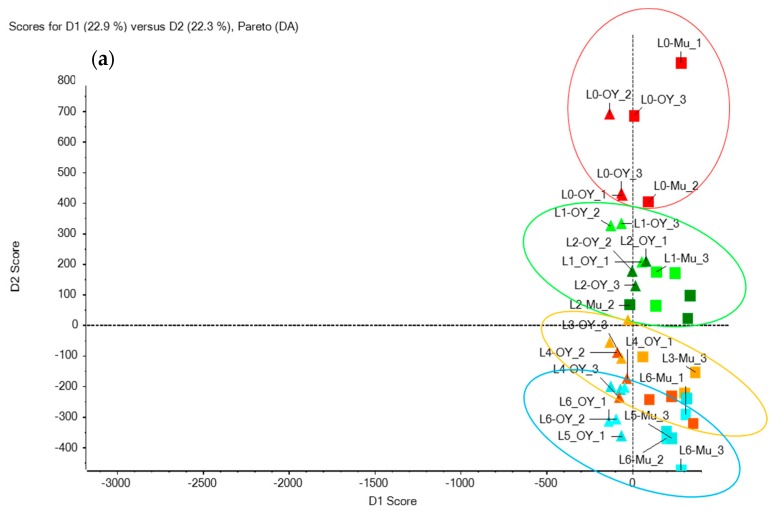

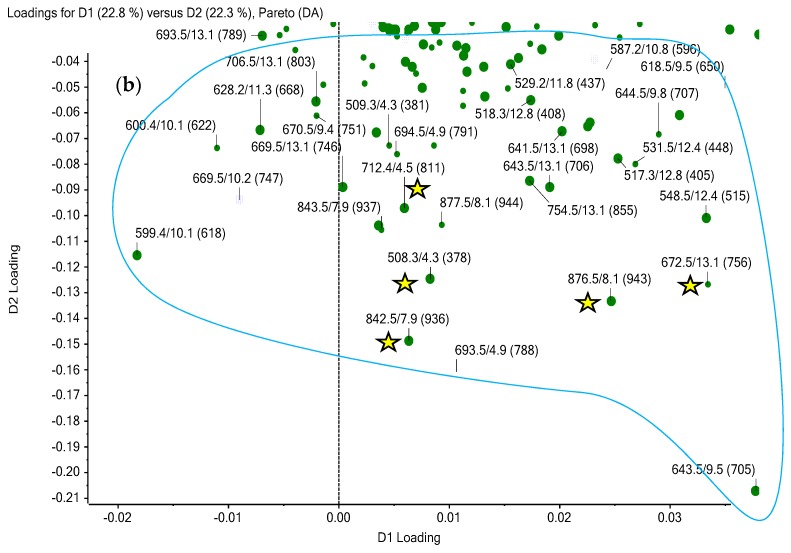
(**a**) Scores plot of a supervised PCA-DA analysis of the data generated after analyzing contaminated and non-contaminated extracts (MeOH, mussel, and oyster) by LC-HRMS in ESI^+^ (in red: L0; green: L1 and L2; orange: L3 and L4; blue: L5 and L6); (**b**) Zoom at the corresponding loadings plot: ions circled in blue are the representative features (green dots) of the most contaminated level (L6). Ions corresponding to the toxins of interest are flagged with yellow stars. The three figures reported next to each green dot (such as 508.3/4.3 (378)) represent, respectively, the exact mass, the retention time, and the feature’s area (in brackets).

Figure 5a shows all the “L0” corresponding to uncontaminated samples are well grouped at the top of the scores plot and separated from the remaining contaminated samples. The other samples are classified according to their concentration levels from the least concentrated to the most concentrated ones. Despite this stratification, the different toxin levels are not clearly separated after the PCA–DA data treatment. This could be explained by the fact that concentrations between levels were close. To identify the ions responsible for the clusterization presented in the loadings plot (Figure 5b), the authors selected those circled in blue as likely to be representative of the most contaminated levels; this included a total of 70 features reduced to 55 when removing the isotopes. The 55 selected features were further refined by excluding those not corresponding to actual peaks; this led to 38 and 36 features for the oyster and mussel matrices, respectively. Using each of the retained features, a tentative formula was generated in Peakview^®^ while using Formula Finder. The number of suggested formulae was highly variable and ranged from 3 to 419 for the features in the oyster samples and from 3 to 426 in the mussels. The toxins were not necessarily among the first proposals in Formula Finder; as an example, PnTX-A was the fourth out of 169 proposed formulae in the mussel extracts, while AZA1 was 8 out of 357. The next step of the general workflow was to upload each generated formula in the ChemSpider database to identify the corresponding compound(s), knowing that several potential compounds could be proposed for each formula. Once a compound was identified, its mol file was downloaded to compare the theoretical (in-silico) and the experimental spectra, provided that a spectrum had been acquired in TOF MS/MS. Following all of this workflow, the toxins (GYM, SPX1, AZA1, PnTX-A, and PTX2) marked with a star in Figure 5b were among the features that were identified as being responsible for the clusterization.

## 3. Discussion

Several papers deal with the characterization/validation of methods for the analysis of targeted marine toxins in low [11,23,32] and high resolution [16,17,25,33,34,35], but there is no report in the literature of the proper characterization of the entire workflow for the non-targeted analysis of these compounds. More generally, there are no internationally recognized guidelines for the validation of non-targeted analysis [36], but tentative validation strategies were undertaken in the field of environmental pollutants [28,31,37]. The common feature between these studies lies in the fact that the validation procedure was carried out for known compounds or metabolites. The rationale behind this approach is that a validated targeted method is an essential step toward the production of a reliable and acceptable data set through the non-targeted approach. Furthermore, the compounds that are targeted should cover a range of polarity as wide as possible, from hydrophilic to lipophilic. This explains the choice of the 18 toxins that were used as part of this study.

### 3.1. Targeted Analysis

The LC-HRMS method developed enabled the separation and the analysis of the 18 marine toxins tested. The resolution of the mass spectrometer (QTOF) enables the unambiguous identification of the toxins. Initially, the HSS T3 column was selected, because it can withstand 100% water as mobile phase and it is indicated for the analysis of polar molecules, such as DA.

According to the criteria of the Commission Decision 2002/657/EC [38] and the associated cutoff values, the method developed gave satisfactory performances for the 14 marine toxins that were selected for the characterization study and spiked in the mussel and oyster matrices. Regarding the case of DA, positive ionization is mostly reported in the literature [39,40,41,42,43], but in this study’s conditions, better sensitivity was observed in ESI^−^, as also reported by Ciminiello et al. [44]. The exact masses for the intra-day and inter-day precision, ranging from 0.4 to 1.7 ppm for the different toxins, showed good stability in mass measurements in this study’s analytical conditions and are consistent with previously published mass accuracy data in LC-HRMS [16,18,45]. The sensitivity of the method was overall satisfactory and estimated LOQs are comparable to previous studies [25,34,39,46]. Regarding YTXs, LODs were relatively higher than expected, as compared to the triple quadrupole detector, but still were considered as acceptable, since they are well below the regulatory threshold of 3.75 mg/kg [5]. Concerning PbTx-2 and 3, even if it has been shown that MeOH improves the sensitivity of the method (by a factor of 6–10) when compared to ACN, in agreement with previous studies, the LOQs obtained under the current conditions remained below the threshold concentration of 800 µg PbTx-2 eq/kg defined in both the American and Australian legislations [32,47].

Concerning the matrix effects, the toxins were affected to different extents with either ion enhancement or ion suppression of different magnitudes. DA was the compound that was the most affected with +33% ion enhancement. To overcome such interferences, matrix-matched calibration curves might be a good solution, as reported elsewhere [11,48]. Sample treatments including purification steps such as solid phase extraction (SPE) or liquid-liquid extraction (LLE) could be effective in removing, or at least reducing the matrix effects but, in non-targeted analysis sample treatment should be kept as simple as possible to avoid losing potential compounds of interest. The matrix effects observed for the compounds spiked into the tested matrices and determined via targeted or suspect analysis can only define a degree of uncertainty for further evaluation [28]. Vergeynst et al. [31] developed a method, including a large volume injection (LVI) to avoid laborious sample enrichment and selective preconcentration of pharmaceuticals in surface waters. The use of a divert valve to eliminate highly polar organic and inorganic (salts) compounds at the beginning of the chromatographic run made the matrix effects comparable to those that were obtained with methods while using SPE as a sample treatment [31]. Dilution could also be advised to reduce the matrix effects, but, in the case of non-targeted analysis, there is a risk of reducing the intensity of the features of interest [28].

When dealing with a chemical method, such as LC–MS, there are several aspects, other than matrix effects, which are likely to impact the method performances. This has been largely reported in the literature [49,50,51,52] and it feeds the controversy upon the replacement of the mouse bioassay, used as a reference method for the analysis of certain toxins, with LC-MS. Although factors such as the availability and stability of standards and reference materials, as well as the difference in sensitivity of the MS analyzers, contribute to the gaps identified in food safety control for marine toxins by chemical methods, the non-targeted approach comes as an answer to one of the major criticisms of LC-MS methods: the analysis of pre-assigned masses, which hinders the detection of emerging and unknown toxins.

### 3.2. Non-Targeted Analysis

The suspect screening approach gives the ability to screen a large list of compounds and to do a retrospective analysis [17,28]. The list that was used as part of this study was composed of 821 compounds including both marine toxins and cyanotoxins with their exact masses. Using suspect screening no standard is required as the identification capability lies on different criteria, among which are the exact *m*/*z* ratio, the isotopic profile, the MS/MS fragmentation pattern.

Different parameters were selected and optimized for accurate mass matches through the suspect list, based on the experience acquired when using the suspect screening approach. As an example, the imposed minimum signal-to-noise ratio was set to 6:1 as a peak picking criterion to determine the decision limit that defines a peak. Nürenberg et al. [28] reported the same value while Krauss et al. went for a value of 5:1 [26]. Overall, based on the chosen criteria the authors defined a traffic light color-coding filtering step (retaining only ‘green light’ features) that was successfully applied to test samples and enabled identifying the marine toxins that were spiked into the blind samples. Although this process was automated, it is important to keep a critical eye on the data generated and to check it to avoid errors.

To allow for confirmation purposes, the experimental fragmentation that was obtained for the toxin tentatively identified was compared to the built-in MS/MS spectrum in the library (when available) or to the in-silico fragmentation pattern that was obtained from a mol file (PubChem, ChemSpider). Regarding the case of in-silico fragmentation, it was necessary to be sure of the quality of the data available from the websites queried to avoid any misidentification.

Following the different optimized steps of the suspect strategy developed, tentative identification of emerging compounds was possible with a high confidence level. The interpretation of fragmentation patterns of HRMS/MS spectra was a successful way to elucidate the structure of a molecule, even in the case of isomeric structures provided that they had significant fragmentation patterns. To unequivocally identify the molecular structure of a compound, further analysis by nuclear magnetic resonance might be needed.

Using the non-targeted screening approach, two different options were tested for the data treatment. The first one consisted of performing a pairwise *t*-test between contaminated (spiked) and non-contaminated samples. This required that an appropriate reference sample be available. It should have the same elemental composition as the contaminated samples, except for the presence of toxins, to make sure that the significant difference that was picked by the statistical test was related to the contaminants and not to the difference in matrix composition. This condition is difficult to meet as there are many environmental factors that are likely to influence the matrix composition. Regarding the case of shellfish for instance, this could be the genus and species of the animal, its age, its geographical origin, the seasonality etc. To circumvent this difficulty in choosing the right reference, an approach consists of creating a library for each type of matrix that would apprehend the diversity in matrix composition mentioned previously. This approach was put in practice by the EURL for pesticides in fruit and vegetables to determine the matrix signature of different foodstuffs belonging to the eight groups that are defined by DG SANTE [53].

The key issue in the data treatment was to reduce the number of relevant features to screen. When using the *t*-test, this can be done with the *p*-value for which different cutoff values can be selected: *p*-value < 0.05 or *p*-value < 0.01. The cutoff value of 0.01 decreased the number of features from 5000 to a number ranging from 20 to 52 depending on the sample, in the current study. This value was too restrictive and some of the spiked toxins could not be found in the final list. A *p*-value of 0.05 was a better compromise, which reduced the final list of features that was composed of 100 to 150 ions without excluding compounds of interest. Mondeguer et al. [35] applied the same strategy and managed to drastically reduce the number of features for different sets of naturally contaminated mussel samples containing AZAs or unknown compounds from the Arcachon bay.

The *p*-values obtained in the pairwise comparison of the samples spiked with the different toxins at different concentrations and the blank sample illustrated the notion of the discriminating power of the statistical analysis. Therefore, depending on the toxins, the statistical test will be more or less efficient in picking the statistical differences between the sets of samples compared. This has nothing to do with the analytical sensitivity of the method. Using a non-targeted screening approach, it is necessary to have both a sensitive method and the appropriate statistical test with a good discriminating power.

Another data treatment option was tested: the multivariate analysis using a PCA or PCA and discriminant analysis (PCA–DA). The PCA was not appropriate as it discriminated the samples (spiked and blank) according to the nature of the matrix rather than according to the toxin composition (Figure S3). The PCA–DA gave a better clusterization of the samples based on the toxin composition by forcing the statistical test to not consider the matrix as a major discriminating factor (Figure 5). Even in PCA–DA, the identification of the features that were responsible for the clusterization of the samples, and likely to explain the differences in composition, is still time and labor intensive and requires a good methodology. Yet, the authors managed to identify the spiked marine toxins in the PCA–DA loadings plot as features potentially explaining the clusterization of the high toxin levels.

Despite the automation of several tasks of the workflow, some of them must be manually done. This is the case, for instance, for the identification of false positive and false negative features that require a visual inspection of the chromatograms [28,37].

Whatever the statistical approach that was chosen, it is necessary to reduce the number of features to facilitate the data treatment and the identification of the compounds of interest. Several options can be adopted: (1) blank exclusion or blank reduction. Using the first case, all of the features present in the blank will be removed from the samples to be analyzed, whereas for blank reduction, the features with intensity in the sample less than ten times higher than in the blank will be excluded. Nürenberg et al. [28] tested blank exclusion and blank reduction and did not see any significant difference. Two types of blanks can be concomitantly used: a procedural blank (solvent) and a blank sample to reduce even further the number of features not related to the contamination event; (2) elimination of the adducts and isomers to reduce further the number of features; (3) limitation of the number of replicates to three as a compromise between a repeatability requirement and the fact that each injection generates its own false positives, thus reducing the proportion of common features between the different injections [28].

Depending on the identification confidence desired and identified by Schymansky et al. [54] as levels one to five, the time needed to perform both the analysis and the data processing could vary significantly. It could take from days for the level 5 (exact mass of interest) to months to reach the level 1 of identification confidence (confirmed structure by reference standard).

The chromatographic conditions in this paper cover a wide range of marine toxins, but they are not the most suitable for specific toxin groups such as palytoxins, ciguatoxins and maitotoxins. However, the same optimized workflow for data treatment can be applied to different extraction and separation methods. The best strategy to enhance hazard identification would be the combination of LC-HRMS with toxicity tests to reduce the size of the search space. Fractions from chromatographic separation containing potential candidates can be collected and screened while using cellular tests to identify the toxic ones. This step allows for focusing analytical efforts on relevant contaminants and ensures the identification of significantly toxic compounds. A similar methodology, while using both cellular tests and mass spectrometry, recently permitted the identification of a novel maitotoxin, MTX-4 [55].

## 4. Conclusions

To assess the potential of the LC-HRMS method to detect marine toxins as part of a non-targeted analysis, the authors performed a proof of concept study as a first essential step toward a reliable characterization of samples naturally contaminated with unknown marine toxins and the identification of the toxins. Since there are no guidelines for the validation of a non-targeted method, the LC-HRMS method that was developed for the analysis of marine biotoxins was characterized according to the approach used in the field of water micropollutants. The method performances were first evaluated in targeted mode for marine toxins with different polarities spiked in mussel and oyster samples and were found to be satisfactory for the criteria tested (LODs, LOQs, specificity, matrix effects, accuracy, and precision). The performances of the optimized non-targeted strategy were then evaluated, both for the suspect screening approach relying on the use of a library of 821 toxins and for the without a priori screening of unknowns. The essential steps for the non-targeted procedure have been detailed and discussed. The overall workflow was tested on spiked samples that were analyzed blindly and was shown to be highly efficient in narrowing down the number of potential false positive and false negative findings. Whatever the approach selected, the marine toxins spiked in the samples analyzed as blind for the proof of concept were picked among the features detected in LC-HRMS. It is important to report that, although many tasks could be automated in the data treatment, it is essential to critically and manually review the results that were obtained to avoid any misinterpretation

As the workflow is time and labor intensive, the number of features should be kept to a minimum by using blanks (procedural and sample) to exclude or reduce the corresponding features, according to the option chosen. The question of the reference sample must be addressed to help in identifying the compounds of interest in the contaminated sample among interfering features; an option could be to create matrix libraries apprehending the wide diversity of the features that are likely to be present in non-contaminated matrices. 

It will be necessary to analyze naturally contaminated samples and to isolate a novel or unknown toxic compound to confirm the efficiency of this methodology. The present study should be completed by testing the non-targeted approach in the ESI^−^ ionization mode while using blind samples spiked with the corresponding toxins. Further developments should be carried out by testing different techniques of extraction, separation, and so on to increase even further the range of the toxins falling into the scope (palytoxin-like, ciguatoxin-like). There is, therefore, a vast area of research on these non-targeted approaches to be investigated in the future to make non-targeted LC-HRMS more powerful for marine toxin monitoring and to guarantee consumer safety.

## 5. Materials and Methods 

### 5.1. Standards and Reagents

All the solutions were prepared with analytical reagent-grade chemicals and ultrapure water (18.2 MΩ cm) produced by purifying deionized water with a Milli-Q Academic water purification system (Millipore S.A., Saint-Quentin-en-Yvelines, France).

Hydrochloric acid (HCl; 37%) and sodium hydroxide (NaOH; 99%) were purchased from Merck (Fontenay-sous-Bois, France). Ammonium formate (>97%) was purchased from Sigma-Aldrich, Saint-Quentin-Fallavier, France. Formic acid (98–100%), acetonitrile (ACN; HPLC grade), and methanol (MeOH; HPLC grade) were purchased from Fisher Scientific SAS (Illkirch, France). Ammonium hydroxide (25%) was purchased from VWR (Fontenay-sous-Bois, France). Atmospheric pressure chemical ionization (APCI) calibration solutions were purchased from Sciex (Nieuwerkerk aan den Ijssel, The Netherlands).

Certified reference materials (CRMs) were purchased from the National Research Council of Canada (NRCC, Halifax, NS, Canada). These included certified calibration solutions of the following toxins: DA, AZA1–3, PTX2, OA, DTX1 and 2, YTX, hYTX, SPX1, PnTX-G, and GYM. Standards of PnTX-A, 13,19-didesMeC, and 20-meG were purchased from Cifga (Lugo, Spain). PbTx-2 and 3 were purchased from Abcam (Cambridge, UK).

### 5.2. Sample Preparation

Blank mussels samples (*n* = 3) and oysters samples (*n* = 3) were prepared according to the standard operating procedure of the EURLMB by extracting 2 g of homogenized tissue with 2 × 9 mL of 100% MeOH [56]. Following centrifugation, the supernatants were combined into a volumetric flask and the volume adjusted to 20 mL while using MeOH.

To detect and quantify the total amount of OA group toxins present, including the esterified forms, an alkaline hydrolysis was performed before LC–MS/MS analysis [57]. Regarding the hydrolysis step, 500 µL of aqueous NaOH 2.5 M solution was added to 4 mL of methanolic extract, homogenised by vortex mixing for 0.5 min and heated at 76 °C for 40 min. Once cooled to room temperature, the extract was neutralised with 500 µL of aqueous HCl 2.5 M solution. Samples were filtered (0.45 µm) prior to analysis.

### 5.3. Preparation of Standards and Matrix-Matched Calibration Solutions

A toxin mixture stock solution was prepared in MeOH from the certified calibration solutions and contained PTX2, AZA1 to 3, OA, DTX1 and 2, PnTX-A, PnTX-G, YTX, h-YTX, SPX1, 13,19-didesMeC, 20-meG, GYM-A, and DA at concentrations ranging from 120 to 240 ng/mL depending on the toxins. This stock solution was serially diluted in MeOH to prepare six working solutions (L1–L6), each containing the studied toxins at different concentrations. Brevetoxins (PbTx-2 and 3) working solutions were prepared separately while using a 250 ng/mL stock solution. These working solutions were then used to prepare matrix-matched standards with previously prepared blank mussel and oyster extracts to reach the appropriate concentration levels: 450 µL aliquots of shellfish extracts were dispensed into HPLC vials, and 50 µL of working solution was added, resulting in six different concentration levels per matrix. This operating procedure resulted in a consistent matrix concentration of 0.09 g/mL at each concentration level. Matrix-free standards were prepared similarly, while using pure MeOH instead of shellfish extracts.

The calibration curves for matrix effect assessments ranged from 1 to 12 ng/mL for AZAs and cyclic imines; 2–24 ng/mL for YTXs, OA, DTXs and PTX2; 6–72 ng/mL for DA.

MeOH and matrix-matched calibration curves, mean slopes, intercept and determination coefficients (*R*^2^) were calculated based on triplicate injections of seven concentration levels (including the blank, L0).

### 5.4. LC-HRMS Analysis

Measurements were carried out by LC-HRMS. A Dionex Ultimate 3000 HPLC system (Thermo Fisher Scientific, San Jose, CA, USA) was coupled to a QTOF (Sciex 5600 Triple TOF, Darmstadt, Germany). The QTOF system was equipped with a DuoSpray ion source and a TurboIonSpray^TM^ probe. The chromatographic separation was achieved on a Waters (Saint–Quentin–en–Yvelines, France) Xselect^®^ HSS T3 column (100 × 2.1 mm, 2.5 µm) with a binary mobile phase of (A) water and (B) ACN–water (95/5, *v/v*), each containing 50 mM formic acid and 2 mM ammonium formate. The gradient of the LC method was composed by the following steps within a total run time of 20 min. Subsequent to an isocratic step for 1 min, a linear gradient was applied from 2% to 100% B within 9 min, and held at 100% of B for 5 min. The initial conditions were reached again and were kept constant for 5 min to re-equilibrate the column. The flow rate was 0.45 mL/min and the column temperature was 30 °C. The injection volume was set to 5 µL.

Concerning the MS detection, electrospray ionization (ESI) was used in positive and negative modes in separate runs. The parameters for positive and negative ionization were as follows (deviating values for negative ion mode are indicated in parentheses): ion source gas (GS) 1 and 2, 35 and 45 psi; curtain gas (CUR), 30 psi; source temperature (TEM), 500 °C; ion spray voltage floating (ISVF), 5.5 (−4.5) kV; declustering potential (DP), 60 V (−100 V); ion release delay (IRD), 67 ms; ion release width (IRW), 25 ms.

The MS was operated in full scan TOF MS and MS/MS modes with information dependent acquisition (IDA) in a single run analysis for targeted and non-targeted screening. The full scan experiment (100–1250 Da) was performed with an accumulation time of 0.2 s while using the high sensitivity mode. An additional eight MS2 spectra experiments (accumulation time: 0.05 s) were programmed. A collision energy spread (CES ± 20 eV) was applied in conjunction with the CE (40 eV) for IDA mode to perform both low and high collision-energy, simultaneously resulting in valuable fragmentation information for identification purposes. The mass spectrometer was recalibrated automatically after five measurements while using an automated calibrant delivery system (CDS) via the atmospheric pressure chemical ionization (APCI) probe of the DuoSpray ion source.

Each sample/standard solution was injected in triplicate to generate enough data to perform the chemometric processing (e.g., *t*-test), as descibed in the next section.

### 5.5. Post-Acquisition Data Processing

The data acquisition was carried out by Analyst^®^ TF 1.7.1 software (Sciex, Toronto, ON, Canada). Data were then processed following three different approaches: (1) quantitative target analysis with reference standards; (2) suspect screening without reference standards; and, (3) non-target screening of unknowns. A diagram of the processing data strategy is shown in Figure 1.

The MasterView^TM^ application of the PeakView^®^ 2.2 software (Sciex, Toronto, ON, Canada) was used to create target and suspect compound lists and display identification criteria while using “traffic lights” on the basis of confidence settings for the following parameters: molecular formula, accurate mass (mass error), isotopic pattern, and MS/MS library (purity score), as well as further peak information, such as retention time, S/N (signal-to-noise ratio), or FWHM (full width at half maximum) (Supplementary Materials Figure S4). The traffic light turns green when the confidence settings of the above-mentioned parameters are met; this indicates a good confidence level in the identification of the compounds in the suspects list.

The quantitation of target toxins was achieved by MultiQuant^TM^ 2.1.1 (Sciex, Toronto, ON, Canada). The decision of whether a feature was counted as a peak was done manually by visual control while using the integrated MultiQuant^TM^ data sets of each XIC (extracted ion chromatogram). Decision criteria for a peak to be recognized as such were the peak shape (approximatively Gaussian), an S/N above 6, and a peak width at the base below 0.6 min.

Concerning the non-target screening purpose, the extraction and the alignment of the features from the full scan experiment were processed by MarkerView^TM^ software 1.2.1 (Sciex, Toronto, ON, Canada). Statistical data analyses (*t*-test, principal components analysis (PCA)) were also performed while using this software. ChemSpider and PubChem databases were used for searching for possible structure identities and MS/MS fragment ion prediction to identify compounds and to characterize chemical structures.

### 5.6. Method Performance Characteristics for Target Quantitation

To assess the method performances and matrix effects, each concentration level of calibration curves was injected in triplicate, alternating between standards in methanol, standards in oyster matrix and standards in mussel matrix. Detection and quantification limits (LOD and LOQ) were first estimated as equivalent to an S/N of 3 and 10, respectively, by analyzing low level spiked extracts in triplicate. Regarding the characterization study, LODs and LOQs were determined with the ordinary least-squares regression data method [58,59] while using solvent and matrix-matched calibration curves. LODs and LOQs were calculated, respectively, as 3 and 10 times the standard deviation of the y-intercepts, over the slope of the calibration curve.

The suitability of the quantitation method for the studied toxins was evaluated following the EU Commission Decision 2002/657/EC as a guideline.

To determine the repeatability and the intermediate precision of the method, mussel samples that were spiked with marine toxins were extracted and injected twice daily, at three different days over the course of two weeks.

The relative standard deviation (RSD) was determined in repeatability (RSD_r_) and within-laboratory reproducibility (RSD_R_) conditions.

Samples provided by the EURLMB as part of proficiency testing schemes for lipophilic toxins and DA were analyzed by LC-HRMS after being extracted as described in Section 5.2., Z-scores [60] were determined for each toxin in the different samples, while using the following equation:

$$\;Z\;score=\frac{x-X}{\sigma }\;$$

with

*x* = analytical result
*X* = assigned value as determined by the EURLMB
*σ* = standard deviation
|z|<2: results are satisfactory
2<|z|<3: results are questionable
|z|>3: results are unsatisfactory

### 5.7. Non-Targeted Screening of Marine Toxins

The workflow was tested by treating a selected set of target compounds (from different toxin families; GYM, SPX1, AZA 1, PnTX A, and PTX2) spiked in MeOH and shellfish extracts (mussel and oyster) at 6 different concentration levels L1–L6 (2, 4, 8, 12, 16, 24 ng/mL) as unknowns to check the performance of the procedure. Blind samples were analyzed in triplicate in ESI^+^. Suspect screening data were treated using PeakView^®^ and MasterView^TM^ softwares, via an XIC list of 821 molecules, including marine toxins and cyanotoxins kindly provided by Dr. A. Gerssen (Rikilt Institute of food safety, Wageningen, The Netherlands). The only a priori information was the exact mass of the protonated ions [M + H]^+^ or [M + NH_4_]^+^ of the toxins that were included in that list. The authors chose to test the workflow in positive mode only.

To contrast suspect screening, the unknown screening strategy was run without any a priori information. Data were processed using MarkerView^TM^ and the workflow included alignment, peak detection, deconvolution, component intensity comparison, and statistics. Two statistical approaches were applied; (1) *t*-test to determine if statistically significant differences between contaminated and non-contaminated samples could be associated to the presence of toxins; and, (2) multivariate statistical analysis (PCA) either supervised or not.

## Figures and Tables

**Table 1:** Limits of quantification (LOQs) of the different toxins in methanol (MeOH) and matrix (mussel and oyster).

	LOQ (µg/kg)		
	MeOH	Mussel	Oyster
AZA1	4.3	4.6	4.5
AZA2	2.7	4.4	3.2
AZA3	5.6	7.6	6.5
PTX2	6.7	8.7	8.4
GYM-A	1.1	2.0	1.6
SPX1	1.7	3.1	2.2
PnTX-A	4.4	5.1	4.6
PnTX-G	5.0	4.4	4.6
13,19-didesMeC	3.3	4.7	4.1
20-meG	3.5	5.0	4.0
OA	6.0	8.9	5.6
DTX1	3.6	4.4	5.6
DTX2	4.2	5.4	5.0
DA	26	30	31
YTX *	87	119	132
hYTX *	84	121	128
PbTx-2 *	280	312	324
PbTx-3 *	300	321	337

* These toxins were not part of the characterization study for sensivity issues. Corresponding LOQs were estimated in a preliminary study, as equivalent to an S/N of 10, by analyzing in triplicate low level spiked extracts.

**Table 2:** Accuracy and precision (*n* = 6) for the quantitative procedure in mussel matrix.

Toxins	Spike Level (µg/kg)	Recovery (%)	RSDr (%)	RSD_R_ (%)
AZA1	10	96	13.7	17.1
	120	105	3.4	5.8
AZA2	10	86	8.2	11.3
	120	100	1.3	4.2
AZA3	10	103	12.9	19.8
	120	104	2.9	6.9
PTX2	20	110	9.4	11.0
	240	101	4.2	4.2
GYM	10	97	6.1	6.1
	120	100	7.3	7.3
SPX1	10	94	3.9	9.1
	120	103	1.6	1.7
PnTX-A	10	90	8.0	8.9
	120	97	4.8	6.2
PnTX-G	10	103	11.4	14.7
	120	95	11.4	14.7
13,19-didesMeC	10	91	8.7	12.1
	120	105	4.1	4.1
20-meG	10	88	9.1	14.1
	20	102	1.7	1.9
OA	20	106	5.9	8.7
	240	103	8.0	9.2
DTX1	20	108	11.9	11.9
	240	106	3.0	3.2
DTX2	20	106	6.9	7.1
	240	104	7.6	8.3
DA	60	99	12.2	12.2
	720	99	1.4	2.7

**Table 3:** *p*-values obtained in a *t*-test comparing pairwise a blank mussel sample with samples spiked at different concentration levels. Non-significant results (*p*-values > 0.05) are indicated in bold and italics.

Spike Levels	SPX1	GYM	AZA1	PnTX A	PTX2
L0/L1	<0.01	***>0.05***	***>0.05***	***>0.05***	***>0.05***
L0/L2	<0.01	<0.01	***>0.05***	***>0.05***	***>0.05***
L0/L3	<0.01	<0.05	<0.01	<0.01	***>0.05***
L0/L4	<0.01	<0.01	<0.01	<0.01	<0.05
L0/L5	<0.01	<0.01	<0.01	<0.01	<0.01
L0/L6	<0.01	<0.01	<0.01	<0.01	<0.01

## Supplementary Materials

The following are available online at http://www.mdpi.com/2072-6651/10/9/375/s1, Figure S1: Mass variations (mass-to-charge-ratio) for the toxins (positive and negative ion mode), Figure S2: Example of tentative identification of non-target compound, Figure S3: Scores plot of a PCA analysis of the data generated after analyzing contaminated and on-contaminated extracts (MeOH, mussel, and oyster) by LC-HRMS in ESI^+^, Figure S4: Example of result display of the MasterView software using “traffic lights” and selected confidence setting for target compounds identification, Table S1: Chemical formula, detected ion, measured mass, *m*/*z* (*n* = 15) and retention time for each toxin in mussel extracts obtained on 5600 Q-TOF, Table S2: Determination coefficients (*r*^2^) for both solvent and matrix-matched calibration curves.
