# Supplementary material for: Extended Targeted and Non-Targeted Strategies for the Analysis of Marine Toxins in Mussels and Oysters by (LC-HRMS)
Source: Toxins (Basel). 2018 Sep 14;10(9):375. doi: 10.3390/toxins10090375 (PMC6162736; doi:10.3390/toxins10090375)
Supplement: Supplementary file 1 [file toxins-10-00375-s001.pdf]

Supplementary Materials: Extended Targeted and Non-Targeted Strategies for the Analysis of Marine Toxins in Mussels and Oysters by LC-HRMS

Inès Dom, Ronel Biré, Vincent Hort, Gwenaëlle Lavison-Bompard, Marina Nicolas and Thierry Guérin

Table S1. Chemical formula, detected ion, measured mass, m/z (n = 15) and retention time for each toxin in mussel extracts obtained on 5600 QTOF. (Mass accuracy and precision expressed by mass errors and SD expressed as ppm are presented in Figure S1).

Toxin	Formula	Detected Ion	Measured m/z	Rt (min)
GYM	C32H45NO4	$[M + H]^+$	508.34199	5.2
PnTX-A	C41H61NO9	$[M + H]^+$	712.44159	5.4
PnTX-G	C42H63NO7	$[M + H]^+$	694.46766	6.2
AZA1	C47H71NO12	$[M + H]^+$	842.50422	8.9
AZA2	C48H73NO12	$[M + H]^+$	856.52089	8.5
AZA3	C46H69NO12	$[M + H]^+$	828.48848	9.2
SPX1	C42H61NO7	$[M + H]^+$	692.45141	5.7
13,19-didesMeC	C41H59NO7	$[M + H]^+$	678.43677	5.3
20-meG	C43H63NO7	$[M + H]^+$	706.46795	5.8
PTX2	C47H70O14	$[M + NH_4]^+$	876.51096	8.8
PbTx-2	C50H70O14	$[M + H]^+$	895.48384	10.0
PbTx-3	C50H72O14	$[M - H]^+$	897.43994	9.1
DA	C15H21NO6	$[M - H]^-$	310.12961	3.5
OA	C44H68O13	$[M - H]^-$	803.46169	7.5
DTX1	C45H70O13	$[M - H]^-$	817.4762	8.6
DTX2	C44H68O13	$[M - H]^-$	803.46169	7.9
YTX	C55H82O21S2	$[M - H]^-$	1141.47295	9.2
hYTX	C56H84O21S2	$[M - H]^-$	1155.48869	9.2

Table S2. Determination coefficients (R^2) for both solvent and matrix-matched calibration curves.

**Toxin**	**Concentration Range (μg/kg)**	**R^2**		
		**MeOH**	**Mussel**	**Oyster**
AZA1	10–120	0.9961	0.9939	0.9965
AZA2	10–120	0.9963	0.9920	0.9938
AZA3	10–120	0.9953	0.9946	0.9919
PTX2	20–240	0.9959	0.9927	0.9948
GYM-A	10–120	0.9973	0.9981	0.9988
SPX1	10–120	0.9972	0.9935	0.9946
PnTX-A	10–120	0.9979	0.9966	0.9968
PnTX-G	10–120	0.9981	0.9974	0.9949
13,19-didesMeC	10–120	0.9974	0.9962	0.9969
20-meG	10–120	0.9970	0.9958	0.9977
OA	20–240	0.9975	0.9966	0.9982
DTX1	20–240	0.9982	0.9990	0.9969
DTX2	20–240	0.9917	0.9926	0.9906
DA	60–720	0.9930	0.9931	0.9905

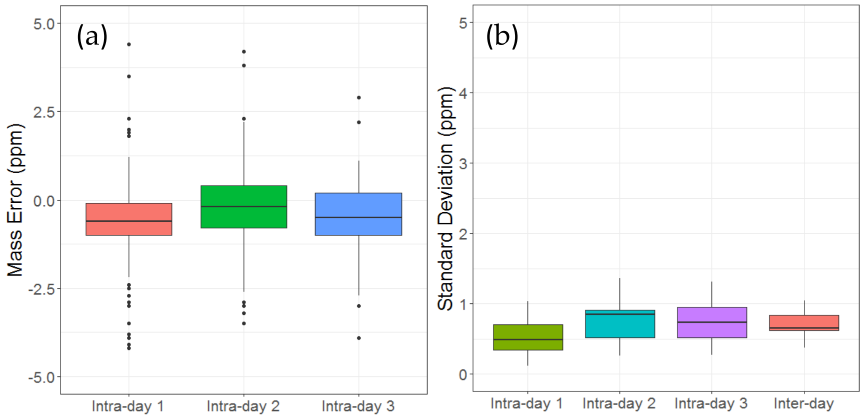

Figure S1. Mass variations (mass-to-charge-ratio) for the toxins (positive and negative ion mode); measurment of the replicates of spiked mussel extracts (five-times injections (inter-day) at three different dates (inta-day)). (**a**): Boxplot of the mass errors related to the theoretical exact mass. (**b**): Boxplot of the intra-day and inter-day precision (SD) of the mass error.

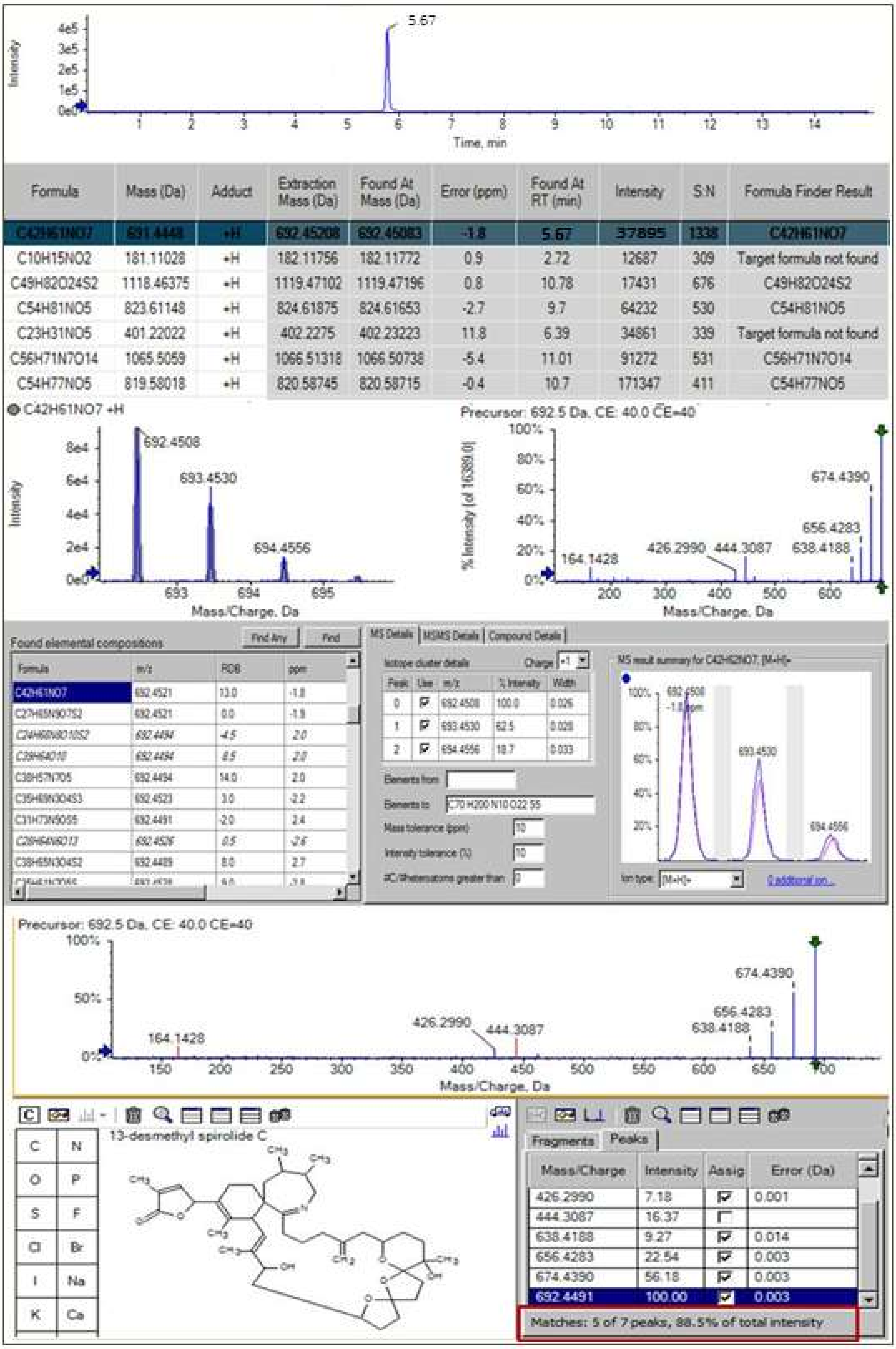

Figure S2. Example of tentative identification of non-targeted compound; identification of 13-desmethyl spirolide was achieved by generation of theorical chemical formula based on accurate mass, isotopic distribution using formula finder. Elemental formula is screened through ChemSpider database to generate possible structures. Proposed molecular structures were checked by comparing experimental MS/MS fragment spectra to theorical fragmentation.

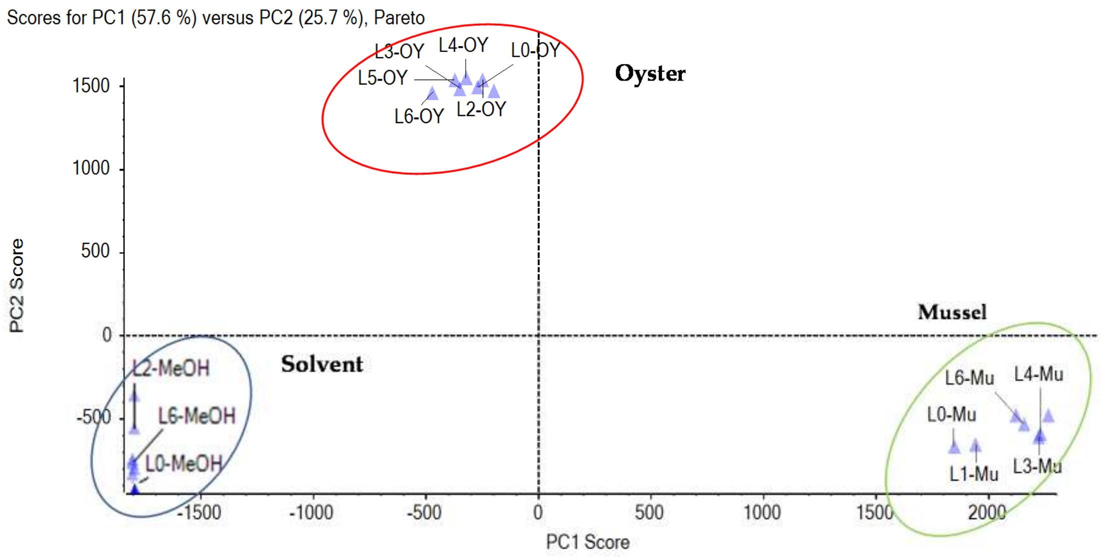

Figure S3. Scores plot of a PCA analysis of the data generated after analyzing contaminated and non-contaminated extracts (MeOH, mussel, and oyster) by LC-HRMS in ESI$^+$.

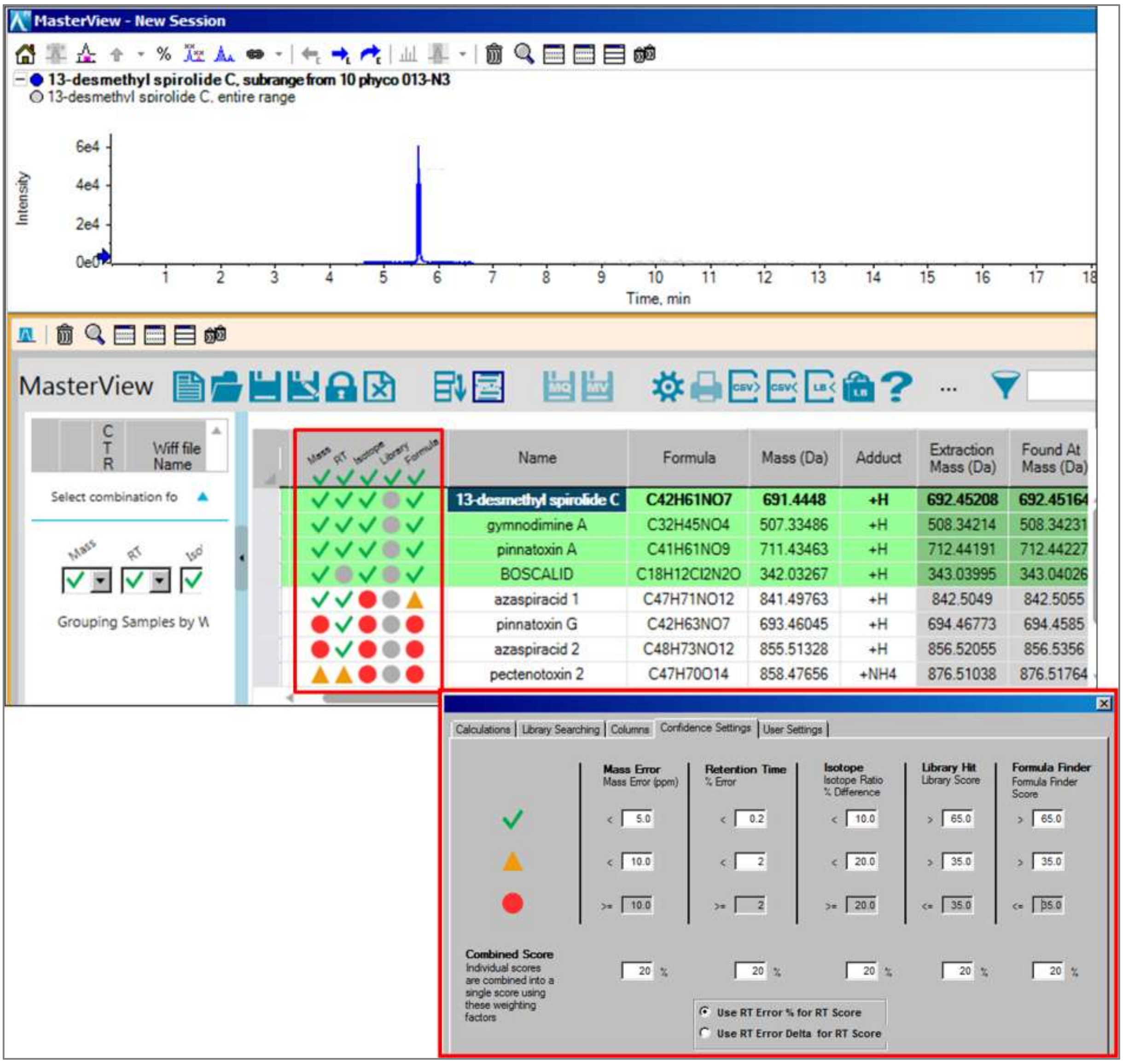

Figure S4. Example of result display of the MasterView software using "traffic lights" and selected confidence setting for target compounds identification.
